# Acoustic difference in advertisement calls among two sympatric *Boulenophrys* species: A confirmatory case to acoustic niche hypothesis and morphological constraint hypothesis

**DOI:** 10.1002/ece3.11318

**Published:** 2024-04-23

**Authors:** Tuo Shen, Jing Liu, Xiujun Tang, Caichun Peng, Shize Li, Chaobo Feng, Lang Mu, Haijun Su

**Affiliations:** ^1^ College of Forestry Guizhou University Guiyang China; ^2^ Biodiversity and Nature Conservation Research Center Guizhou University Guiyang China; ^3^ Department of Resources and Environment Moutai Institute Renhuai China; ^4^ Leigongshan National Nature Reserve Administration Bureau of Guizhou Leishan China; ^5^ College of Life Sciences Guizhou University Guiyang China

**Keywords:** acoustic differences, acoustic niche hypothesis, *Boulenophrys*, morphological constraint hypothesis, sympatric species

## Abstract

In anurans, acoustic communication is the most important form of communication at the interspecific and intraspecific levels. Acoustic diagnostic features may be a potential alternative to morphometric and molecular diagnostics. Here, we assessed the variations in advertisement calls between two sympatric species, *Boulenophrys leishanensis* and *Boulenophrys spinata*, that share their breeding season and breeding sites. In addition, we investigated any potential relationships between call parameters and body size. We found that the advertisement calls of both species are simple calls. The two species exhibited significant differences in all call parameters. Both *B. leishanensis* and *B. spinata* showed a significant negative correlation with their body size on dominant frequency. These differences in call parameters may play an important role in interspecific recognition. Additionally, because intraspecific acoustic variation reflects body size, calls may be relevant for sexual selection. Our study supports the acoustic niche hypothesis and the morphological constraint hypothesis and calls are a valid tool for distinguishing between the two species of *Boulenophrys* in the field.

## Graphical abstract

We assessed the variations in advertisement calls between two sympatric species, *Boulenophrys leishanensis* and *Boulenophrys spinata*. In addition, we investigated any potential relationships between call parameters and body size. Our study provides an effective case for the acoustic niche hypothesis and the morphological constraint hypothesis.

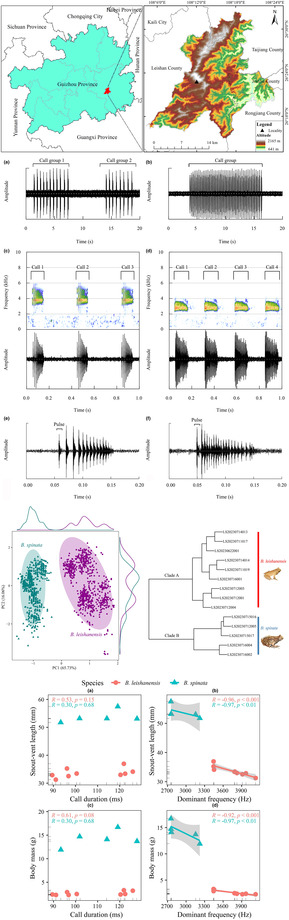

## 1 INTRODUCTION

Acoustic communication is an important way of animal communication and has been extensively studied in mammals, birds, bats, insects, and amphibians (Fan et al., 2022; Kingston & Rossiter, 2004; Luo & Huang, 2021; Shen et al., 2023; Wu et al., 2023). Calling is a critical mode of interspecific and intraspecific interaction in anurans, where it is crucial for interspecific identification, reproduction, and evolution (Brenowitz & Rose, 1999; Cunningham & Birkhead, 1998; Kelley, 2004). To further understand the relationship between frog behaviors and vocal communication, researchers have divided frog calls into four types: feeding calls, aggressive calls, reproductive calls, and defensive calls. Advertisement calls, courtship calls, amplectant calls, release calls, post‐oviposition male release calls, and rain calls are examples of reproductive calls (Köhler et al., 2017; Toledo et al., 2015). Furthermore, advertisement calls exhibit substantial variation across species, rendering them a viable foundation for the systematic classification and identification of cryptic species (Sullivan et al., 1996).

Closely related species that coexist in sympatry often require similar resources, including food, habitat, breeding space, and time (Chesson, 2000). Consequently, the possibility of interspecies mating exists among congeneric species that coexist in the same site (Köhler et al., 2017). However, the phenomenon of hybridization enhances the risk of interspecies mating simultaneously, which ultimately fails reproduction (Dodd Jr., 2013; Gerhardt & Huber, 2002). Species must employ a variety of reproductive strategies to overcome this problem, such as isolation in breeding space and time, as well as differences in reproductive behavior (Chen et al., 2020; Wang et al., 2019). Acoustic niche hypothesis suggests that if there is no significant difference in call parameters between coexisting closely related species, they will not reproduce at the same time period (Duellman & Pyles, 1983). In complex and resource‐scarce environments where several species are under pressure to breed at the same time, differences in advertisement calls enable species‐specific recognition of acoustic signals (Köhler et al., 2017; Ryan & Wilczynski, 1988). As a result, sympatric species using similar breeding habitats can remain isolated through differing acoustic properties, thereby avoiding breeding failure (Forlani et al., 2013). The phenomenon of acoustic niche partitioning has been reported not only in insects and birds but has even been substantiated in fossilized organisms (Chitnis et al., 2020; Sueur, 2002; Xu et al., 2022).

Previous studies have shown that morphological constraints generally seem to play a pervasive role in the evolution of animal acoustic communication, and proposed the morphological constraint hypothesis. (Mikula et al., 2020; Ryan & Brenowitz, 1985). A negative relationship between body size and frequency of acoustic signals seems to be a general rule in animal bioacoustics (Gillooly & Ophir, 2010; McClatchie et al., 1996; Pearse et al., 2018). In anurans, the spectral and temporal parameters of calls could reflect frog body size (Liu et al., 2018; Wang et al., 2012; Wei et al., 2012). However, other studies suggest that acoustic parameters and morphological characteristics are unrelated in some frog species (Márquez et al., 2005; Penna, 2004; Wang et al., 2021).

*Boulenophrys* Fei, Ye, & Jiang, 2016 (Anura: Megophryidae) is a genus with 67 species that are distributed from South China, Vietnam, Laos, Thailand, and Myanmar (Frost, 2024). Among them, 61 species of *Boulenophrys* are currently known from Southeast China (Amphibia China, 2024). The *B. leishanensis* and the *B. spinata* were first described in 2020 and 1973, respectively (Hu et al., 1973; Li et al., 2018). The type localities of the *B. leishanensis* and the *B. spinata* are both located in Leishan County, Guizhou Province, China. Previous studies mainly focused on taxonomy and phylogeny of two species (Chen et al., 2017; Lyu et al., 2023; Mahony et al., 2017). There have been scattered reports on the acoustics of these two species (Li et al., 2018; Su et al., 2020), but limited samples allowed only a few simple analyses of call parameters and structure. However, no comprehensive comparison of call parameters has been made between the two sympatric species thus far. Is there a call isolation between two species? And do their calls reflect body size? Although they have been discovered for many years, the above issues are still unknown. The absence of this information hinders the identification of these two sympatric species in the field.

The study aimed to (1) compare the spectral and temporal parameters of advertisement calls from sympatric *B. leishanensis* and *B. spinata* to test acoustic niche hypothesis for the two species; (2) evaluate the relationships between the body size of signalers and the call parameters of their advertisements calls to determine whether species of *Boulenophrys* support the morphological constraint hypothesis.

## 2 MATERIALS AND METHODS

### 2.1 Vocalization recording and analyses

Our experimental procedures complied with the applicable laws on animal welfare and research in China and were approved by the Subcommittee on Experimental Animal Ethics of Guizhou University (Permit No. EAE‐GZU 2023‐E013).

**FIGURE 1:**
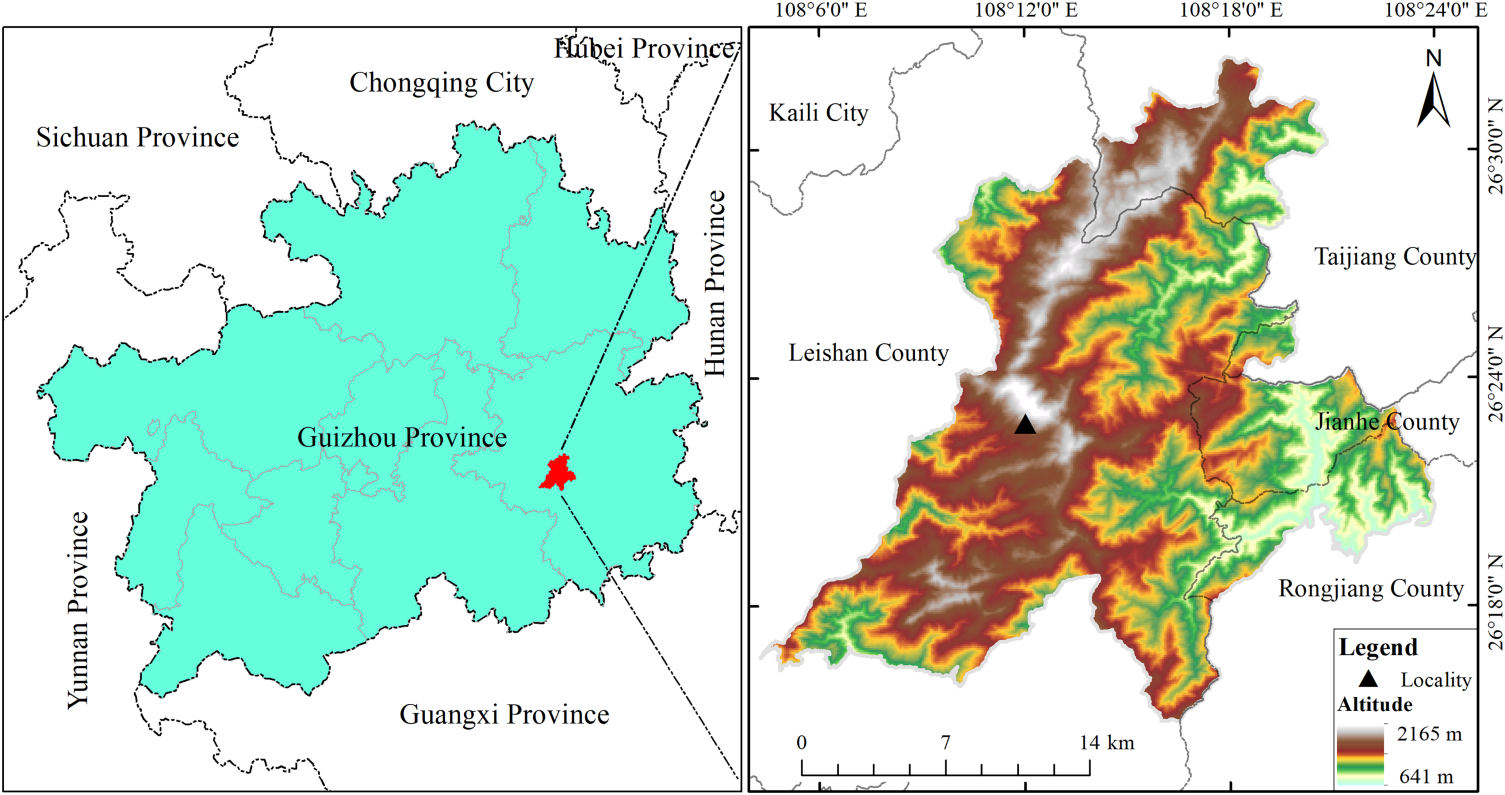
Geographical location of the two sympatric species of *Boulenophrys*, Leishan County, Guizhou Province, China.

The advertisement calls of *B. leishanensis* and *B. spinata* were both recorded from their type localities Leishan County, Guizhou Province, China (Figure 1). Calls of each individual were obtained using a digital recorder, SONY ICD‐PX470 (sampling rate 44.1 kHz, 16‐bit resolution). Each call was recorded within a 0.5 m distance from the calling individual. Recorded calls were always of isolated individuals and never from a mixed chorus. The recordings were saved as WAV files. The advertisement call characteristics were analyzed with the software Raven Pro 1.6. (K. Lisa Yang Center for Conservation Bioacoustics at the Cornell Lab of Ornithology, 2023). Temporal properties were measured using Raven's waveform display. Spectral properties were measured by averaging the spectrum over the entire duration of a call (Hann window, DFT = 512 samples, overlap = 50%, and Hop Size = 256 samples). Only calls that had a high signal‐to‐noise ratio and were free from overlapping calls of nearby males were used for the analysis. We used “call‐centred” terminology as summarized by Köhler et al. (2017), in which the fundamental unit was defined as a “call” and the continuous units were defined as a “call group.” We measured all parameters and characteristics following the procedure described by Köhler et al. (2017) and Qian et al. (2023), including (1) call duration (ms), CD; (2) call intervals, CI; (3) number of calls per call group, NCC; (4) number of pulses per call, NPC; (5) pulse rate (pulses/s), PR; (6) dominant frequency (Hz), DF; (7) frequency 5%, fre 5%; and (8) frequency 95%, fre 95%. Oscillograms and spectrograms were generated using Seewave v.2.2.0 (Sueur et al., 2008) and TuneR 1.4.2 (Ligges et al., 2013) packages in R program 4.2.2 (R Core Team, 2021) with a “Hanning” window size of 256 samples and an overlap of 50%. Descriptive statistics of call characteristics, mean, standard deviation (SD), and range, were computed using SPSS 23.0.

### 2.2 Morphological data

To compare the morphological characteristics of the two species, the animals were captured by hand after their calls were recorded. Their body mass (BM) was measured using an electronic balance, and 17 morphological indices, such as snout–vent length (SVL), head length (HDL), head width (HDW), snout length (SL), internasal distance (IND), upper eyelid width (UEW), interorbital distance (IOD), eye diameter (ED), maximal tympanum diameter (TYD), tympanum–eye distance (TEY), length of lower arm and hand (LAL), hindlimb length (HLL), thigh length (THL), tibia length (TL), maximal tibia width (TW), length of foot and tarsus (TFL), and foot length (FL), were measured using a digital caliper. Measurement standards were based on studies by Li et al. (2020) and Fei et al. (2005).

### 2.3 Statistical analyses

Prior to analysis, we tested all the data for assumptions of normality and homogeneity of variance using the Shapiro–Wilk and Levene's tests, respectively. We compared the call parameters of the two species using the Mann–Whitney *U* test.

We compared the variability among call parameters by determining within‐individual and among‐individual coefficients of variation (Bee et al., 2016; Pettitt et al., 2013).

$$\mathrm{CV}=\mathrm{SD}/X^{*}100\%.$$

For each call parameter, we first calculated the individual mean (Xw) and standard deviation (SDw). Then, the grand mean and standard deviation were determined. After that, the within‐individual coefficient of variation (CVw) and among‐individual coefficient of variation (CVa) were calculated.

$$\mathrm{CVw}=\mathrm{SDw}/{\mathrm{Xw}}^{*}100\%.$$

$$\mathrm{CVa}=\text{grand}\;\mathrm{SD}/\text{grand}\;X^{*}100\%.$$

Based on the scheme of Gerhardt (1991), we classified call parameters as static (CVw < approx. 5%), dynamic (CVw > approx. 12%), and intermediate (CVw 5%–12%). We calculated the ratio of among‐male to within‐male coefficients of variation (CVa/CVw) as a measure of relative among‐male variability (Feng et al., 2009; Robisson et al., 1993). If CVa/CVw > 1.0 for a call trait, there is more variability among males and this may have behavioral consequences for individual recognition (Forti et al., 2016).

To test for significant differences in call parameters of the two species, we performed a principal component analysis (PCA) based on seven call variables extracted from 1421 advertisement calls: 752 *B. leishanensis* calls emitted by night individuals and 669 *B. spinata* calls emitted by five individuals. We plotted a scatter plot to graphically represent the relationship between the two species from the two principal components retained. In order to test the results of PCA, a hierarchical clustering using Mahalanobis distance was conducted (Mahalanobis, 1936). The dendrogram was constructed based on Ward's method (Ward Jr, 1963). Spearman correlation analysis was used to determine whether dominant frequency and call duration were associated with body size. All statistical analyses were conducted in R 4.2.2 (R Core Team, 2021).

## 3 RESULTS

### 3.1 Call characteristics

**FIGURE 2:**
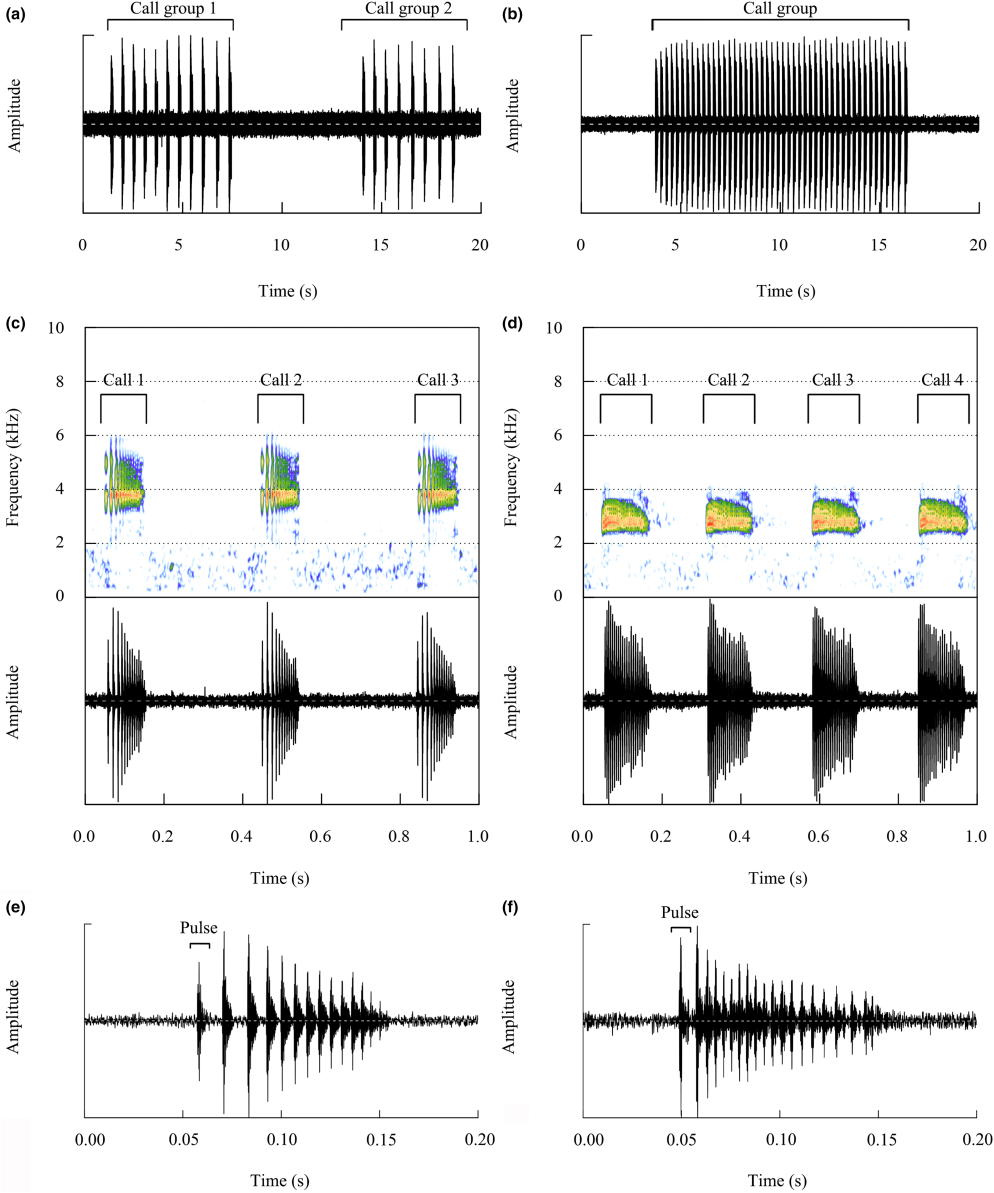
Advertisement calls of the two *Boulenophrys* species. (a) 20 s oscillograms showing two call groups of *Boulenophrys leishanensis*. (b) 20 s oscillograms showing one call group of *Boulenophrys spinata*. (c) 1 s oscillograms and corresponding spectrograms showing three calls of *B. leishanensis*. (d) 1 s oscillograms and corresponding spectrograms showing four calls of *B. spinata*. (e) 0.2 s oscillograms showing one call of *B. leishanensis*. (f) 0.2 s oscillograms showing one call of *B. spinata*.

Both the advertisement calls for *B. spinata* and *B. leishanensis* were simple calls. We recorded and analyzed the spontaneous vocalizations of 752 calls from nine *B. leishanensis* males (ca. 1790 m elev., 15–28°C air temperature, and 90%–94% ambient humidity). Males were observed calling on rocks in streams surrounded by shrubs and forests. The advertisement call of *B. leishanensis* was a group of repeated pulsative calls (Figure 2e). Call amplitude was consistent across all call groups, with the exception of the initial 1–2 calls, which exhibited a lowered amplitude. The initial call commenced with a moderate amplitude within each call group, followed by a distinct interval. The amplitude modulation of the second call increased suddenly, followed by a gradual increase to its peak amplitude at approximately half of the way through the call group (Figure 2a,c). The mean call duration of *B. leishanensis* was 107.47 ± 15.82 ms. The mean intercall interval was 392.10 ± 101.21 ms. The mean pulse number was 15.00 ± 3.00, with a mean pulse rate of 139.27 ± 26.95 pulses/s. The mean dominant frequency was 3728.68 ± 288.49 Hz. The acoustic properties of the spectral and temporal structures of *B. leishanensis* are shown in Table 1.

**TABLE 1:** Advertisement call parameters of *Boulenophrys leishanensis* and *Boulenophrys leishanensis*.

Call parameters	*B. leishanensis*		*B. spinata*		*p*‐Value
	Mean ± SD	Min–max	Mean ± SD	Min–max	
Call duration (ms)	107.47 ± 15.82	47.90–133.80	112.37 ± 13.06	72.5–136.9	.000
Call interval (ms)	392.10 ± 101.21	227.83–961.29	143.56 ± 36.26	98.72–638.01	.000
No. of pulses per call	15.00^a^ ± 3.00^b^	7.00–23.00	24.00^a^ ± 1.50^b^	16.00–31.00	.000
No. of calls per call group	13.67 ± 4.29	4–22	41.81 ± 5.59	30–48	.000
Pulse rate	139.27 ± 26.95	65.18–229.65	207.23 ± 33.59	151.98–325.00	.000
Dominant frequency (Hz)	3728.68 ± 288.49	3445.31–4134.38	2926.2 ± 297.24	2756.25–3445.31	.000
Frequency 5% (Hz)	2985.33 ± 308.35	2756.25–3445.31	2067.19	2067.19	.000
Frequency 95% (Hz)	4837.18 ± 341.03	4134.38–5857.03	3724.44 ± 338.52	3445.31–4134.38	.000

^a^ Median instead of mean.

^b^ Interquartile range instead of SD.

The spontaneous vocalizations of 669 calls from five *B. spinata* males were recorded and analyzed (ca. 1790 m elev., 24–28°C air temperature, and 90%–99% ambient humidity). The call structure of the *B. spinata* is similar to that of the *B. leishanensis* (Figure 2f). Call amplitude was consistent across all call groups, with the exception of the initial 1–2 calls, which exhibited a lowered amplitude. The second call experiences a sudden increased amplitude modulation, which then gradually increases to reach its peak amplitude at approximately one‐sixth of the way through the call group. After reaching the peak amplitude, it was maintained until the end of the call group (Figure 2b,d). The mean call duration of *B. spinata* was 112.37 ± 13.06 ms. The mean intercall interval was 143.56 ± 36.26 ms. The mean pulse number was 24.00 ± 1.50, with a mean pulse rate of 207.23 ± 33.59 pulses/s. The mean dominant frequency was 2926.2 ± 297.24 Hz. The acoustic properties of the spectral and temporal structures of *B. spinata* are shown in Table 1.

### 3.2 Individual variation in call parameters

The descriptive statistics and estimates of variability for all the call parameters are presented in Table 2. We categorized the dominant frequency, frequency 5%, and frequency 95% as static traits for *B. leishanensis* based on the CVw values; the call interval and number of calls per call group as dynamic traits; and the call duration, number of pulses per call, and pulse rate as intermediate traits. Call parameters that varied more among individuals also tended to vary more within individuals (e.g., call interval and number of calls per call group; Table 2) in *B. leishanensis*.

**TABLE 2:** Measures of within and among individual variability for eight advertisement call properties in *Boulenophrys leishanensis* and *Boulenophrys spinata*.

Call parameters	*B. leishanensis*				*B. spinata*			
	CVa	CVw	Type	CVa/CVw	CVa	CVw	Type	CVa/CVw
Call duration (ms)	14.72	5.83 (3.10–17.98)	Intermediate	2.52	11.62	4.35 (2.54–7.30)	Static	2.67
Call interval (ms)	25.81	19.26 (12.11–30.41)	Dynamic	1.34	25.26	17.14 (7.02–42.03)	Dynamic	1.47
No. of pulses per call	23.86	6.61 (4.44–10.46)	Intermediate	3.61	9.97	5.60 (4.54–7.07)	Intermediate	1.78
No. of calls per call group	31.38	23.38 (9.19–60.33)	Dynamic	1.34	13.37	9.85 (1.24–16.44)	Intermediate	1.36
Pulse rate	19.35	7.18 (3.51–18.89)	Intermediate	2.69	16.21	4.86 (3.66–6.26)	Static	3.33
Dominant frequency (Hz)	7.74	2.62 (0.00–9.03)	Static	2.95	10.16	4.62 (0.00–10.82)	Static	2.20
Frequency 5% (Hz)	10.33	3.58 (0.00–11.22)	Static	2.89	0.00	0.00	Static	0.00
Frequency 95% (Hz)	7.05	4.46 (0.00–7.76)	Static	1.58	9.09	4.09 (0.00–7.46)	Static	2.22

The static characteristics attributed to *B. spinata* were the call duration, pulse rate, dominant frequency, and frequency of 5% and 95%, and the call interval was categorized as a dynamic trait. As intermediate characteristics, the number of pulses per call and the number of calls per call group were categorized. Call parameters that varied more among individuals also tended to vary more within individuals (e.g., call interval; Table 2). The frequency of 5% exhibited the lowest variation both among individuals (CVa) and within individuals (CVw). However, the pulse rate had higher CVa/CVw ratios (3.33) than the dynamic properties (1.47) and intermediate properties (range: 1.36–1.78; Table 2). In *B. leishanensis* and *B. spinata*, CVa/CVw > 1.0 was 8 and 7 call parameters, respectively (Table 2).

### 3.3 Determining the difference in advertisement calls between two sympatric species

The Mann–Whitney *U* test indicated that the call duration, number of pulses per call, number of calls per call group, and pulse rate of *B. leishanensis* were significantly lower or shorter than those of *B. spinata* (*p* < .001; Table 1). The call interval, dominant frequency, frequency of 5%, and frequency of 95% of *B. leishanensis* were significantly higher or longer than those of *B. spinata* (*p* < .001; Table 1).

**FIGURE 3:**
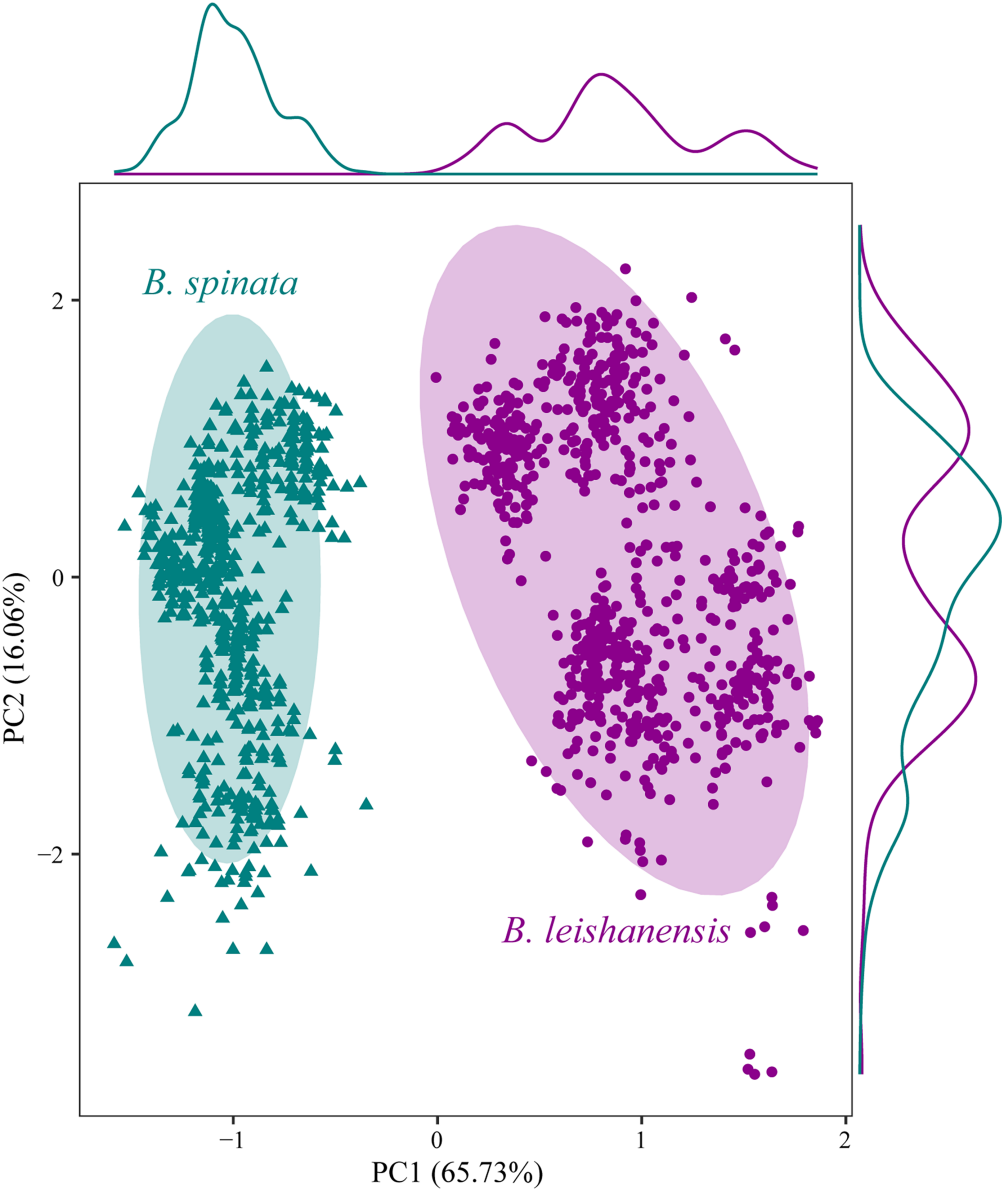
Plots of the first principal component (PC1) versus the second (PC2) for males of *Boulenophrys leishanensis* (purple confidence ellipse) and *Boulenophrys spinata* (green confidence ellipse) resulted from a principal component analysis on seven call parameters.

The results of PCA showed that the difference in call parameters between *B. leishanensis* and *B. spinata* results in two PCs, with eigenvalues values of 4.60 and 1.12, explaining a cumulated variance of 81.79% (Table 3). The confidence ellipses for each PC do not overlap (Figure 3), illustrating the acoustic segregation between the two species.

**TABLE 3:** Principal components and their values resulting from the principal component analysis computed to segregate acoustic properties between the two species.

Call parameters	Principal components	
	1	2
Call duration	−0.29	0.94
Dominant frequency	0.89	−0.09
Freq 5 Hz	0.91	0.03
Freq 95 Hz	0.90	−0.07
No. of pulses per call	−0.88	0.14
Call interval	0.82	0.28
Pulse rate	−0.80	−0.35
Eigenvalue	4.60	1.12
Variance (%)	65.73	16.06
Cumulative variance (%)	65.73	81.79

**FIGURE 4:**
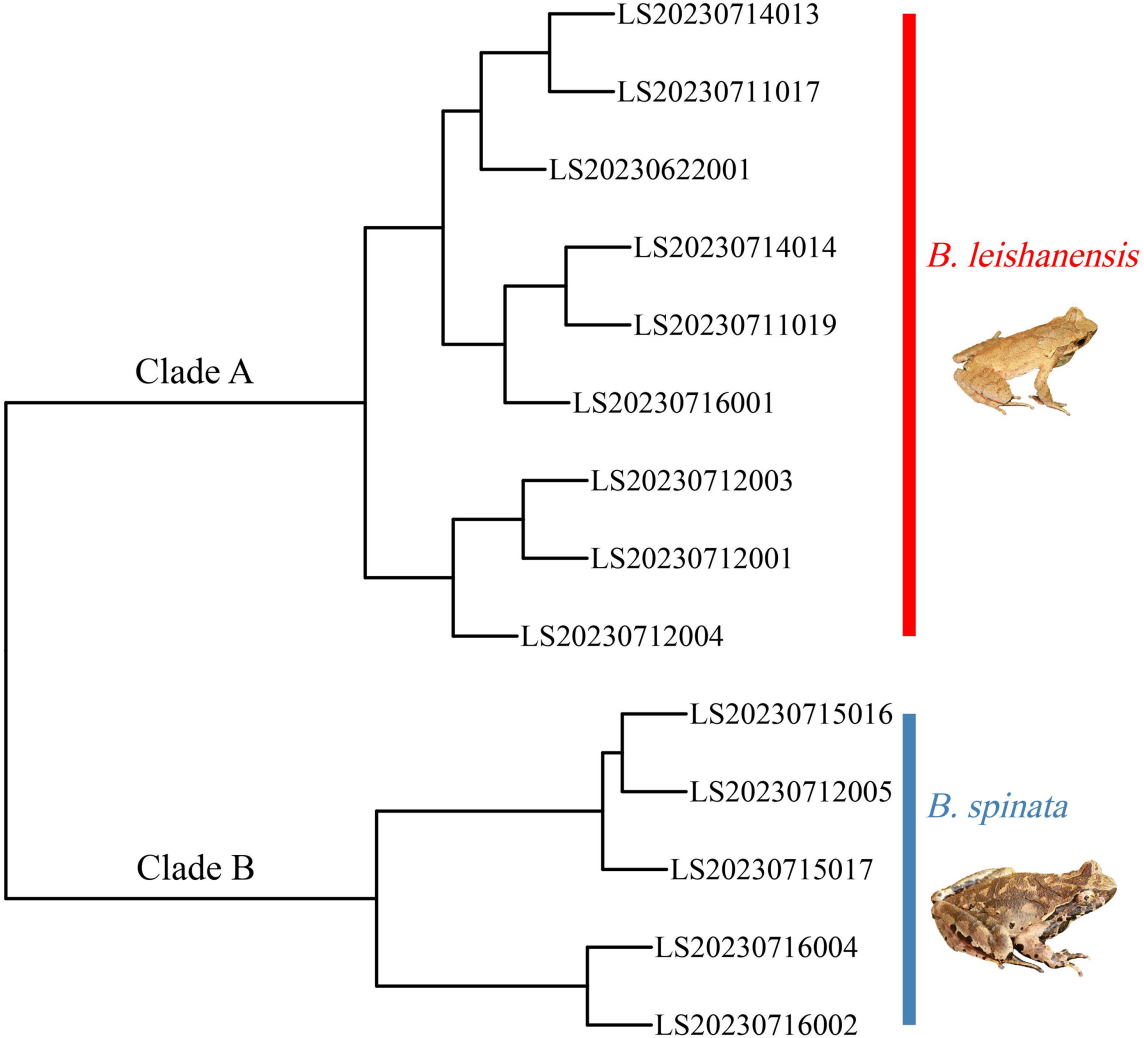
Hierarchical clustering of advertisement calls of *Boulenophrys leishanensis* and *Boulenophrys spinata*.

Fourteen calling males were clustered into two clades based on call parameters of advertisement calls. All male of *B. leishanensis* was assigned to clade A, while all male of *B. spinata* was assigned to clade B (Figure 4).

### 3.4 Relationship between body size and the main call parameters

**FIGURE 5:**
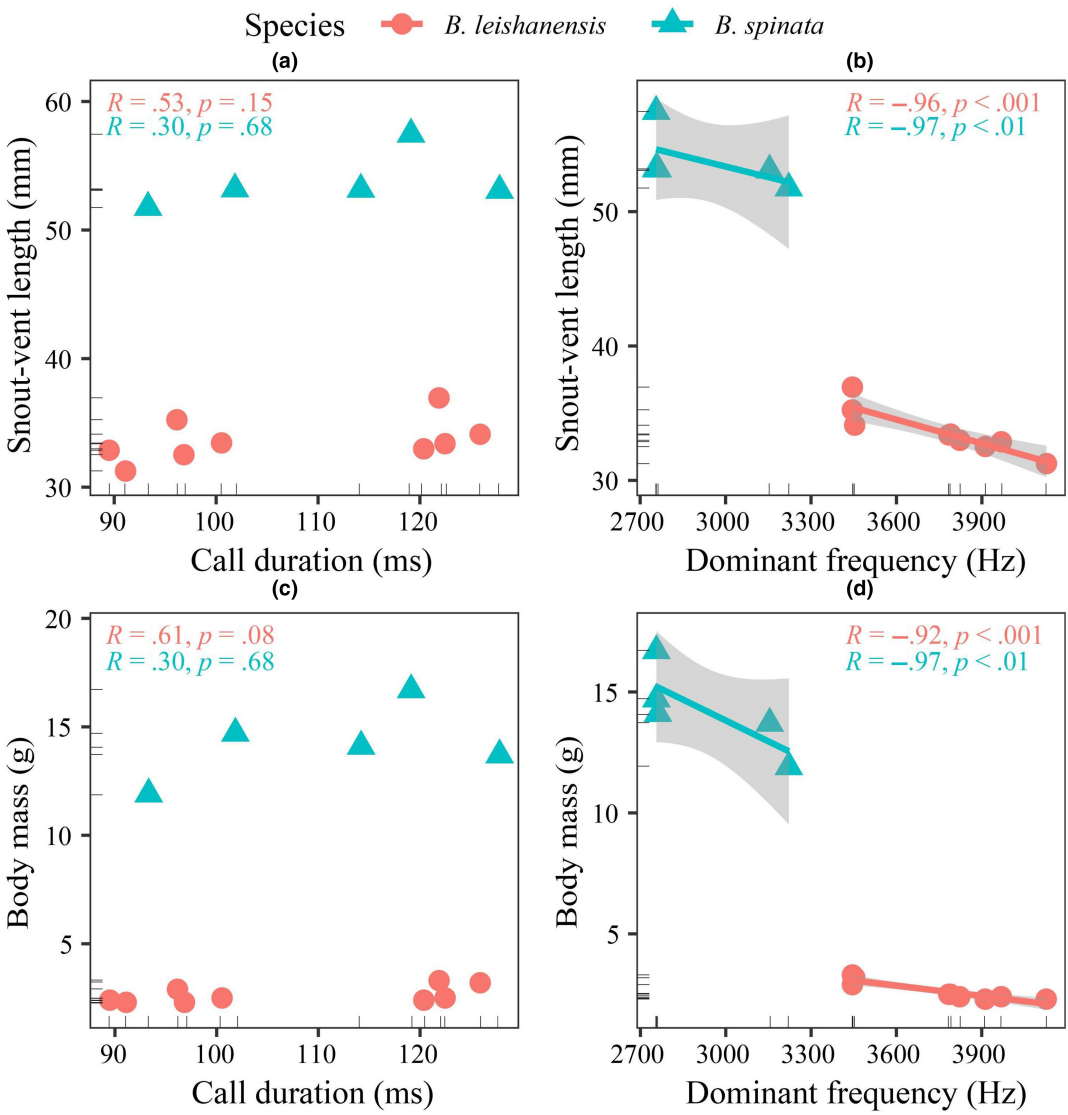
Relationship between call parameters and morphological indices for two species. (a) Relationship between call duration and snout–vent length (SVL). (b) Relationship between dominant frequency and SVL. (c) Relationship between call duration and BM. (d) Relationship between dominant frequency and BM.

The morphological indices were considerably heavier or longer in *B. spinata* males than in *B. leishanensis* males (*p* < .01; Table 4). In *B. leishanensis*, the dominant frequency exhibited a negative correlation with SVL and BM (*R* = −.96, *p* < .001; *R* = −.92, *p* < .001; *n* = 9, respectively) (Figure 5b,d). The dominant frequency was negatively correlated with SVL (*R* = −.97, *p* < .01, *n* = 5; Figure 5b) and BM (*R* = −.97, *p* < .01, *n* = 5; Figure 5d) in *B. spinata*. However, neither species exhibited a significant correlation between body size and call duration (Figure 5a,c).

**TABLE 4:** Morphological indices of male *Boulenophrys leishanensis* and *Boulenophrys spinata*.

Morphological index (g or mm)	*B. leishanensis*		*B. spinata*		*p*‐Value
	Mean ± SD	Min–max	Mean ± SD	Min–max	
BM	2.64 ± 0.39	2.3–3.3	14.22 ± 1.74	11.90–16.70	.003
SVL	33.64 ± 1.65	31.25–36.94	53.73 ± 2.17	51.75–57.45	.003
HDL	9.63 ± 0.55	8.99–10.92	14.25 ± 0.15	14.03–14.42	.003
HDW	10.60 ± 0.51	9.87–11.27	17.91 ± 0.76	16.87–18.73	.003
SL	4.02 ± 0.22	3.66–4.38	6.21 ± 0.27	5.90–6.50	.003
IND	3.48 ± 0.33	3.02–3.98	5.47 ± 0.28	5.09–5.80	.003
UEW	3.13 ± 0.40	2.60–4.07	4.67 ± 0.31	4.21–5.06	.003
IOD	3.18 ± 0.26	2.88–3.59	4.63 ± 0.21	4.46–4.98	.003
ED	3.41 ± 0.37	2.95–3.86	5.62 ± 0.41	5.05–6.16	.003
TYD	2.28 ± 0.29	1.88–2.64	2.92 ± 0.28	2.45–3.21	.009
TEY	2.41 ± 0.25	2.10–2.84	3.32 ± 0.40	2.91–3.89	.003
LAL	15.60 ± 0.57	14.93–16.36	24.22 ± 0.79	23.16–25.26	.003
LW	2.46 ± 0.25	1.96–2.70	4.26 ± 0.52	3.64–5.01	.003
HLL	51.57 ± 1.44	49.94–54.33	87.37 ± 4.17	81.19–92.15	.003
THL	14.19 ± 0.79	13.02–15.64	24.35 ± 0.99	23.34–25.96	.003
TL	16.65 ± 0.52	15.51–17.31	28.63 ± 1.25	26.77–29.84	.003
TW	2.87 ± 0.34	2.40–3.34	6.13 ± 0.91	4.90–6.98	.003
TFL	23.42 ± 0.82	21.95–24.82	40.68 ± 1.80	37.93–42.44	.003
FL	14.25 ± 0.50	13.44–15.33	25.27 ± 1.79	22.44–27.27	.003

Abbreviations: BM, body mass; ED, eye diameter; FL, foot length; HDL, head length; HDW, head width; HLL, hindlimb length; IND, internasal distance; IOD, interorbital distance; LAL, length of lower arm and hand; LW, lower arm width; SL, snout length; SVL, snout–vent length; TEY, tympanum–eye distance; TFL, length of foot and tarsus; THL, thigh length; TL, tibia length; TW, maximal tibia width; TYD, maximal tympanum diameter; UEW, upper eyelid width.

## 4 DISCUSSION

Acoustic signals are among the most crucial forms of communication in anurans amphibians (Köhler et al., 2017). The present investigation ascertained that there exist notable distinctions between *B. leishanensis* and *B. spinata* with regard to call parameters during the production of advertisement calls. Based on the described advertisement calls differences, the two species could be easily distinguished in the field.

In genus of the *Boulenophrys*, the phenomenon of sympatric distribution is quite common, and many researchers have previously discussed the issue of acoustic isolation among sympatric species (Chen et al., 2017). For instance, the *Boulenophrys kuatunensis* and the *Boulenophrys baishanzuensis* coexisted in the Wuyi Mountain of China. The two species exhibit similar dominant frequency in their advertisement calls (3.38–3.75 kHz vs. 3.19–3.38 kHz), but the former vocalizes only during the months of April to May while the latter does so exclusively in June to July (Wu et al., 2020). For the two species in this study, despite the *B. leishanensis* and the *B. spinata* sharing breeding habitats and seasons, their call structures are distinct from each other, and the differences in their dominant frequencies are quite significant. This suggests that the two species have generated differentiation in their acoustic niches. This result is consistent with the predictions of the acoustic niche hypothesis (Duellman & Pyles, 1983).

During their breeding season, male *Boulenophrys* frogs emit repeated, simple calls from stream banks and often form small chorus groups (Pope, 1931; Wang et al., 2014). Previous studies have shown that the main role of choruses is to increase attraction to females and reduce the risk of predation (Wijayathilaka & Meegaskumbura, 2016). At the same time, the cost of individual successful reproduction in choruses is higher, so the energy required for calling is also higher. The periodic pauses in call groups and calls by males of *B. leishanensis* and *B. spinata* may be aimed at restoring and preserving energy.

Our results showed that the examined spectral parameters (e.g., dominant frequency, frequency of 5%, and frequency of 75%) were not or weakly variable as their CVw is smaller than 5%, and a temporal parameter (call intervals) was variable as its CVw was larger than 10% in the two sympatric *Boulenophrys* species. These results were similar to those of most of reported 48 anurans, of which the dominant frequency of 69% of species was classified as a static property and call intervals of 40% of species were classified as a dynamic property (Köhler et al., 2017). Static properties are more important in species recognition because females usually prefer the values of individual calls at or near the mean of the population (Pettitt et al., 2013). In contrast, dynamic call properties could reliably indicate a male's competitive and resource‐acquiring abilities (Gerhardt, 1991). In addition, females tend to prefer extreme values of male calls (Bee et al., 2013; Ryan & Keddy‐Hector, 1992).

Certain species exhibit a correlation between call parameters and body size; these characteristics may serve as crucial signals concerning male–male competition and mate selection (Zhu et al., 2016). In this study, we found that the dominant frequency was negatively correlated with body size both in *B. leishanensis* and *B. spinata*, thus corroborating findings from many other anuran species and providing evidence that dominant frequency is a reliable indicator of male body size (Liu et al., 2018; Wang et al., 2012). Although some studies suggested that species morphological characteristics are not related to call parameters (Wang et al., 2021), our results support the morphological constraint hypothesis to some extent (Ryan & Brenowitz, 1985).

*Boulenophrys* is the largest branch of the subfamily of Megophryinae, and most of them are distributed in southern China (Frost, 2024; Lyu et al., 2023). A summary of the major acoustic characters of the advertisement calls of *Boulenophrys* species for which comparable acoustic data are available is presented in Table S1 in order to provide a quick key for future taxonomic research on the genus. So far, advertisement calls for only 29 of the 67 *Boulenophrys* species that have been described (Table S1). In addition, most previous descriptions of *Boulenophrys* species were based on only a few dozen advertisement calls, and mostly belonging to only a single individual, which cannot provide a better understanding of the intraspecific variation. The lack of acoustics data has become an obstacle to research on bioacoustics monitoring (Xiao et al., 2023). Therefore, there is an urgent need to collect and enrich the acoustics data of the *Boulenophrys*.

In summary, our study provides the first detailed analyses of the call parameters of *B. leishanensis* and *B. spinata*, demonstrating acoustic differences between the two sympatric species. Our results provide basic data for further acoustic, taxonomic, and ecological studies in the genus *Boulenophrys*. In addition, our study provides case support for the acoustic niche hypothesis and the morphological constraint hypothesis.

## AUTHOR CONTRIBUTIONS

**Tuo Shen:** Data curation (equal); formal analysis (equal); investigation (equal); methodology (equal); software (equal); writing – original draft (lead). **Jing Liu:** Methodology (equal); software (equal); writing – review and editing (equal). **Xiujun Tang:** Investigation (equal). **Caichun Peng:** Investigation (equal). **Shize Li:** Conceptualization (equal); writing – review and editing (equal). **Chaobo Feng:** Investigation (equal); methodology (equal). **Lang Mu:** Investigation (equal). **Haijun Su:** Conceptualization (equal); methodology (equal); writing – review and editing (lead).

## Supporting information


Table S1.

## Data Availability

The data supporting the findings of this study can be found in Figshare at https://doi.org/10.6084/m9.figshare.24807543.
